# Influence of different position modal parameters on milling chatter stability of orthopedic surgery robots

**DOI:** 10.1038/s41598-024-61362-2

**Published:** 2024-05-08

**Authors:** Heqiang Tian, Bo Pang, Junqiang Liu, Debao Meng, Xiaoqing Dang

**Affiliations:** 1 College of Mechanical and Electronic Engineering, Shandong University of Science and Technology, Qingdao, 266590 China; 2 Qingdao Municipal Hospital Group East Hospital, Qingdao, 266071 China

**Keywords:** Orthopedic surgery robot, Milling chatter, Zero-order frequency domain method, Modal parameters, SLD, Engineering, Biomedical engineering, Mechanical engineering

## Abstract

This research is dedicated to exploring the dynamics of milling chatter stability in orthopedic surgery robots, focusing on the impact of position modal parameters on chatter stability. Initially, we develop a dynamic milling force model for the robotic milling process that integrates both modal coupling and regenerative effects. We then employ the zero-order frequency domain method to derive a chatter stability domain model, visually represented through stability lobe diagrams (SLDs). Through conducting hammer test experiments, we ascertain the robot's modal parameters at varying positions, enabling the precise generation of SLDs. This study also includes experimental validation of the chatter SLD analysis method, laying the groundwork for further examination of chatter stability across different positional modal parameters. Finally, our analysis of the variations in modal parameters on the stability of robot milling chatter yields a theoretical framework for optimizing cutting parameters and developing control strategies within the context of orthopedic surgery robots.

## Introduction

Orthopedic surgery robots are widely used for bone cutting, repair, joint replacement, and spine surgery^1^. However, orthopedic surgery robots face milling chatter challenges when milling bone materials due to improper cutting parameter settings, inadequate mechanical rigidity, weak damping and time-varying dynamic characteristics, unstable or worn tooling, material vibration characteristics, resonance during the cutting process, and unreasonable cutting trajectories^2^. These factors interact to induce unstable vibrations in the robot during milling, negatively affecting bone machining accuracy, patient safety, surgery duration, and tool durability^3^. Extreme cases may lead to tool breakage and bone fractures, harming patients^4^. Studying the stability of robotic bone milling chatter is pivotal for minimizing vibrations during robotic-assisted surgeries, directly contributing to increased precision and reducing the risk of complications. By ensuring stability during bone milling, outcomes can be significantly improved, directly impacting patient safety and recovery.

Tobias et al.^5^ and Tlusty et al.^6^ have classified self-excited chatter into two main types: regenerative chatter and modal coupled chatter. Both types of chatter can occur during robot milling, affecting machining quality and robot stability. Regenerative chatter refers to the phenomenon where cutting forces interact with the robot structure's vibration during machining tasks, resulting in self-excited oscillations within the system. Specifically, the cutting forces generated during the machining process induce vibrations in the robot structure, which subsequently affect the cutting conditions, leading to periodic fluctuations in cutting forces and eventual occurrence of chatter. The characteristic feature of regenerative chatter in robot milling is its association with the vibration frequency of the robot structure. Modal coupling chatter, on the other hand, involves interactions between different vibration modes within the mechanical system during machining tasks, leading to uncontrolled vibration phenomena. Specifically, vibrations in the robot structure and dynamic properties during the machining process may couple with each other, resulting in nonlinear growth of vibrations and eventual onset of chatter. This effect can lead to reduced accuracy, increased tool wear, chatter, and process instability, influenced by factors such as robot structure, cutting parameters, tool characteristics, and cutting materials.

Pan et al.^7^ investigated the influence of regenerative chatter and modal coupled chatter in robotic machining and found that modal coupled chatter tends to occur when the structural stiffness is lower than the machining process stiffness. Siddhpura et al.^8^ concluded that regenerative chatter negatively affects any machining process. Wang et al.^9^ asserted that modal coupled chatter predominates in robot milling due to the low stiffness of orthopedic surgery robots. Gienke et al.^10^ proposed an extended theory of modal coupled chatter applicable to robot cutting, accurately predicting its occurrence. Mejri et al.^11^ combined modal analysis and experimentation to analyze regenerative chatter and proposed a stability prediction method. Iglesias et al.^12^ discussed the challenges associated with chatter-free machining and highlighted the influence of robot stiffness and natural frequency. Zhang et al.^13^ explored the influence of milling parameters on the milling stability of a 6-DOF robot, while Guo et al.^14^ studied the machining chatter mechanism in the boring process of a robot and observed modal coupled chatter occurring before regenerative chatter.

Regenerative chatter and modally coupled chatter are vibration problems closely related to the robot cutting process, they can occur in specific situations and affect the machining results and robot stability. To better understand and address these vibration issues in robot milling systems, it is necessary to consider the system's multi-modal characteristics, which involve interactions among multiple vibration modes^15^. Methods^16,17^ to reflect multi-modal characteristics in dynamic models include modal superposition, modal coupling, finite element method, and modal analysis. These methods effectively capture the multi-modal characteristics in dynamic models, enabling more accurate prediction of system dynamic response and stability.

The stability lobe diagram (SLD) is an effective method to get a clear picture of the modal characteristics of a system, including the number of vibration modes, their frequency distribution, and their interactions for parameter selection and chatter avoidance, with prediction methods primarily including frequency domain and time domain approaches^18^. The zeroth-order frequency domain method^19^ is commonly used to predict the stability region of a system under different cutting parameters, indicating the range of parameter combinations within which the system can maintain a stable machining state. This method offers the advantage of fast computation speed, making it suitable for rapid preliminary assessment of system stability. Semi-discrete methods^20^ combine features of continuous and discrete systems, often discretizing the characteristic equation of the continuous system to assess stability. Fully discrete methods^21^, on the other hand, accurately consider the dynamic response of the system by converting continuous time into discrete time and solving for stability using numerical simulation methods. Semi-discrete or fully discrete methods are more suitable for conducting more precise stability analysis and simulation. However, compared to classical semi-discrete or fully discrete methods, the zeroth-order frequency domain method is typically more simplified and may entail certain approximation errors^22^.

Gonul et al.^23^ determined dynamic parameters of a robot milling system under different poses and used the frequency domain method to predict the SLD. Mejri et al.^24^ employed a single-frequency method to predict the SLD of robot milling and examined the influence of pose changes on milling stability. Li et al.^25^ developed a 2-DOF robot milling dynamics model considering structural modes, enhancing the accuracy of SLD prediction. Du et al.^26^ developed a dynamic model for multi-point contact in robotic milling, accounting for force-induced deformation, regenerative effects, and process damping, and used a fully discrete method to predict chatter stability. Xin et al.^27^ established a stability prediction model considering low-frequency tool tip vibration caused by robot structural modes, emphasizing the impact of different robot poses on stability improvement boundaries.

Scholars have used theoretical analysis and experimental verification to study robot milling chatter stability, gaining insights into regenerative chatter and modal coupled chatter. These studies have contributed to guiding parameter selection, determining system modal parameters, and developing control strategies for improving stability, which provide a foundation and solutions for addressing chatter issues in robot bone milling. This paper aims to establish modal equations for orthopedic surgery robot milling, specifically considering modal effects. We construct a chatter stability domain model using the zeroth-order frequency domain method, draw the SLD, and quickly analyze the influence of modal parameters on chatter stability. Sections include establishing the domain model, identifying modal parameters, verifying the stability diagram, and examining the influence of modal parameters on milling stability. This study aims to contribute to the existing body of knowledge by focusing on modal effects in orthopedic surgery robot milling and providing insights into the influence of modal parameters on chatter stability, using a method based on the zeroth-order frequency domain.

## Modeling of milling chatter stability domain

**Figure 1:**
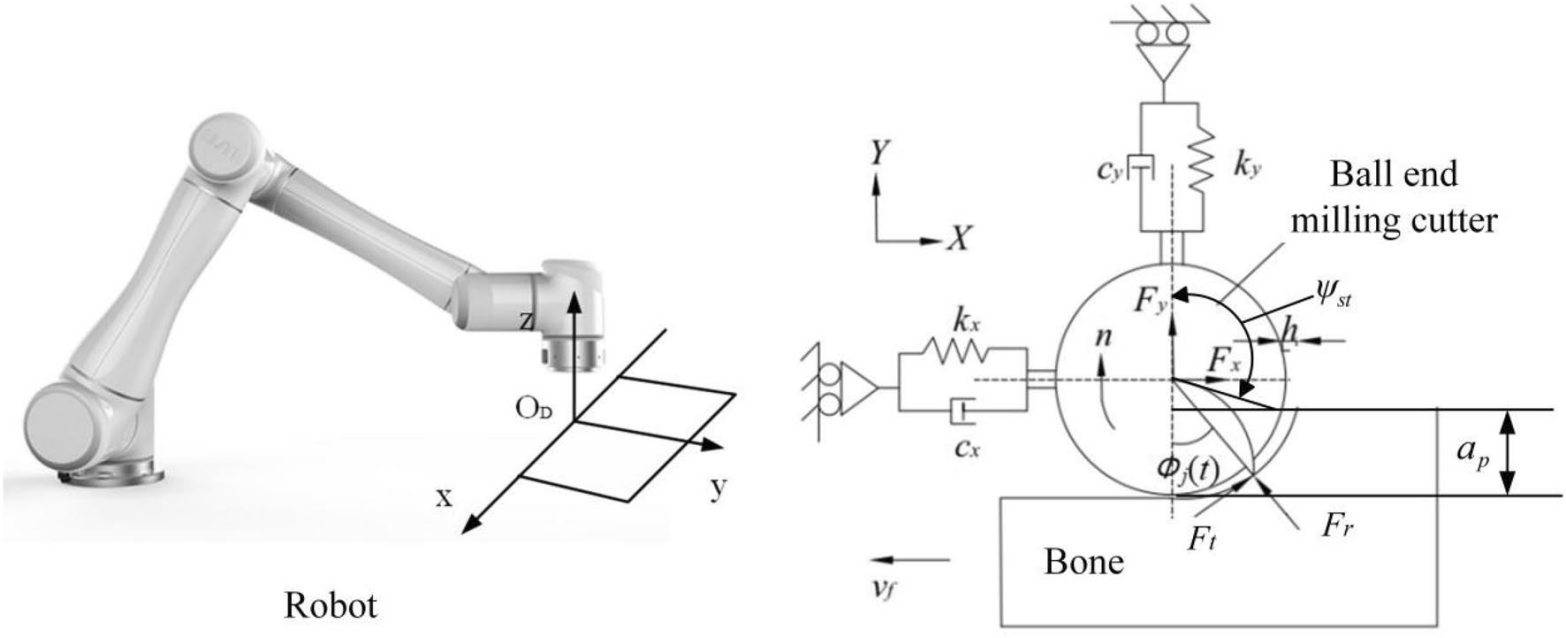
Dynamic milling force model for robot milling system.

During robot-assisted bone cutting and machining, a dynamic system is constituted by the integration of an orthopedic surgery robot, a ball-end milling cutter, and a bone block. This complex system is susceptible to the occurrence of chatter, a phenomenon with profound implications for the accuracy and safety of surgical procedures. Notably, during milling operations executed by the orthopedic surgery robot, chatter is notably exacerbated in the direction perpendicular to the axis of the ball-end milling cutter, while its manifestation along the cutter's axis is comparatively subdued. By considering the modal coupling effect and the regenerative effect, it becomes feasible to formulate a dynamic model governing cutting forces in both the *X* and *Y* directions within the robot milling system, as delineated in Fig. 1. Such a model assumes pivotal significance in comprehending and mitigating chatter, thereby augmenting the efficacy and dependability of robotic-assisted surgical interventions.

The system comprising the robot, ball end milling cutter, and bone block is simplified as a 2-degree-of-freedom (2DOF) vibration system. Each direction, *X* and *Y*, is represented as a spring-damping system, and the dynamic mechanical equations can be expressed as follows:

1 $$\left\{ \begin{gathered} m_{x} \ddot{x}(t) + c_{x} \dot{x}(t) + k_{x} x(t) = F_{x} (t) \hfill \\ m_{y} \ddot{y}(t) + c_{y} \dot{y}(t) + k_{y} y(t) = F_{y} (t) \hfill \\ \end{gathered} \right.$$

where $m_{x}$ and $m_{y}$ represent the equivalent masses of the milling system in the *X* and *Y* directions, respectively. $c_{x}$ and $c_{y}$ are the equivalent damping coefficients of the milling system, while $k_{x}$ and $k_{y}$ represent the equivalent stiffness coefficients of the milling system.

The variables $x(t)$ and $y{(}t{)}$ denote the vibration displacement in the *X* and *Y* directions, respectively, for the ball end milling cutter. Similarly,$\dot{x}{(}t{)}$ and $\dot{y}{(}t{)}$ represent the vibration velocities, while $\ddot{x}(t)$ and $\ddot{y}{(}t{)}$ indicate the vibration accelerations of the ball end milling cutter. The terms $F_{x} {(}t{)}$ and $F_{y} {(}t{)}$ represent the dynamic milling forces acting on the ball end milling cutter in the *X* and *Y* directions, respectively.

The dynamic milling force in the *X* and *Y* directions can be mathematically represented as follows:

2 $$\left\{ {f(t)} \right\} = \left\{ \begin{gathered} F_{x} (t) \hfill \\ F_{y} (t) \hfill \\ \end{gathered} \right\} = \frac{1}{2} \cdot a_{p} \cdot K_{tc} \cdot \left[ {A(t)} \right]\left[ {\Delta t} \right]$$

where $\left[ {A(t)} \right]$ is the dynamic milling force direction matrix of the milling cutter, and $A{(}t{)}$ is a periodic function,$a_{p}$ is milling thickness, *K*_*tc*_ is the tangential milling force coefficient and $\Delta t$ is the milling time.

Applying the Fourier transform to the periodic function $A{(}t{)}$ and omitting higher-order vibration harmonics, we obtain the zero-order prediction function $A_{{0}}$ when the ball-end milling cutter is between the cut-in angle $\psi_{st}$ and cut-out $\psi_{st}$ angle:

3 $$[A_{0} ] = \frac{1}{{\psi_{p} }}\int_{{\psi_{st} }}^{{\psi_{ex} }} {[A(\psi )]{\text{d}}\psi = \frac{N}{2\pi }} \left[ {\begin{array}{*{20}c} {\alpha_{xx} } & {\alpha_{xy} } \\ {\alpha_{yx} } & {\alpha_{yy} } \\ \end{array} } \right]$$

As shown in Fig. 1, $\psi_{st} = 180 - \arccos (\frac{{R - a_{p} }}{R})$,$\psi_{ex} = 180$.

where the average direction coefficient in Eq. (3) is expressed as follows:

4 $$\left\{ \begin{gathered} \alpha_{xx} (t) = \frac{1}{2}\left[ {\cos 2\psi_{j} - 2(K_{rc} /K_{tc} )\psi_{j} + (K_{rc} /K_{tc} )\sin 2\psi_{j} } \right]_{{\psi_{st} }}^{{\psi_{ex} }} \hfill \\ \alpha_{xy} (t) = \frac{1}{2}\left[ { - \sin 2\psi_{j} - 2\psi_{j} + (K_{rc} /K_{tc} )\cos 2\psi_{j} } \right]_{{\psi_{st} }}^{{\psi_{ex} }} \hfill \\ \alpha_{yx} (t) = \frac{1}{2}\left[ { - \sin 2\psi_{j} + 2\psi_{j} + (K_{rc} /K_{tc} )\cos 2\psi_{j} } \right]_{{\psi_{st} }}^{{\psi_{ex} }} \hfill \\ \alpha_{yy} (t) = \frac{1}{2}\left[ { - \cos 2\psi_{j} - 2(K_{rc} /K_{tc} )\psi_{j} - (K_{rc} /K_{tc} )\sin 2\psi_{j} } \right]_{{\psi_{st} }}^{{\psi_{ex} }} \hfill \\ \end{gathered} \right.$$

where *K*_*tc*_ is the tangential milling force coefficient, and *K*_*rc*_ is the radial milling force coefficient.

The radial dynamic milling force of the ball end milling cutter can be determined by combining Eq. (2) and Eq. (3):

5 $$\left\{ {f(t)} \right\} = \frac{1}{2} \cdot a_{p} \cdot K_{tc} \cdot [A_{0} ][\Delta t]$$

The frequency domain representation of the vibration function at the frequency $\omega_{c}$ is given by:

6 $$\left\{ \begin{gathered} \Delta_{t} (i\omega_{c} ) = G(i\omega_{c} )\left\{ {f(t)} \right\}e^{{i\omega_{c} t}} \hfill \\ \Delta_{t - T} (i\omega_{c} ) = e^{{ - i\omega_{c} T}} \Delta_{t} (i\omega_{c} ) \hfill \\ \end{gathered} \right.$$

where $G{(}i\omega_{c} {)}$ is the frequency response function matrix for the contact area between the ball-end milling cutter and the bone.

Then the regenerative dynamic displacement of the ball end milling cutter during milling is expressed as:

7 $$\Delta (i\omega_{c} ) = \Delta_{t} (i\omega_{c} ) - \Delta_{t - T} (i\omega_{c} ) = \left( {1 - e^{{ - i\omega_{c} T}} } \right)e^{{i\omega_{c} t}} G(i\omega_{c} )\left\{ {f(t)} \right\}$$

Substituting Eq. (7) into Eq. (5), we obtain the following equation:

8 $$\left\{ {f(t)} \right\}= \frac{1}{2} \cdot a_{p} \cdot K_{tc} \cdot (1 - e^{{ - i\omega_{c} T}} )\left[ {A_{0} } \right] \cdot G(i\omega_{c} ) \cdot \left\{ {f(t)} \right\} \cdot e^{{i\omega_{c} t}}$$

By solving Eq. (8), the critical axial depth of cut $a_{p\lim }$ can be determined when the chattering frequency $\omega_{c}$ is present:

9 $$a_{p\lim } = - \frac{{2\pi \lambda_{R} }}{{NK_{tc} }}\left( {1 + \kappa^{2} } \right)$$

where $\kappa$ is the ratio of the real part $\lambda_{R}$ to the imaginary part $\lambda_{I}$ of the eigenvalue of the equation.

The formula for $\kappa$ is defined as follows:

10 $$\kappa = \frac{{\lambda_{I} }}{{\lambda_{R} }} = \frac{{\sin (\omega_{c} T)}}{{1 - \cos (\omega_{c} T)}}$$

The corresponding spindle speed $n$ for the critical axial depth of cut can be calculated using the following formula:

11 $$n = \frac{60}{{NT}} = \frac{{60\omega_{c} }}{{N((2k + 1)\pi - 2\arctan (\lambda_{I} /\lambda_{R} ))}}$$

where *T* is the cutter tooth milling period,* N* is the number of cutter teeth, and *k* is the lobe number.

**Figure 2:**
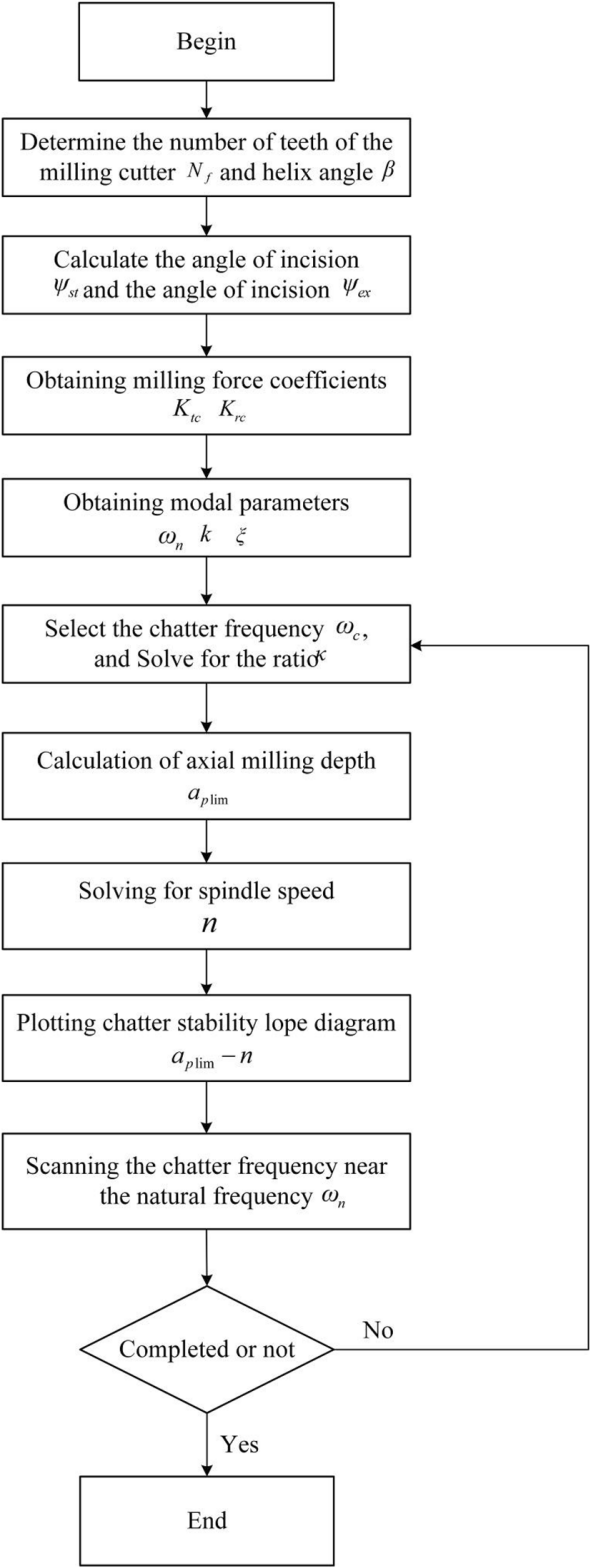
Flowchart illustrating the process of plotting the chatter SLD.

Once the milling force coefficients ($K_{tc}$ and $K_{rc}$) and modal parameters ($\omega_{n}$, $k$ and $\xi$) of the milling system are obtained, the analytical method can be employed to create the robot milling chatter stability lobe diagram. This diagram provides a visual representation of the system's stability boundaries during the milling process. In this diagram, the horizontal coordinate represents the milling spindle speed $n$ and the vertical coordinate represents the axial milling depth $a_{p\lim }$. The specific process for drawing the stability lobe diagram is illustrated in Fig. 2 below.

## Modal parameter identification experiment

### Milling force coefficient identification experiment

To calculate the milling stability domain and construct the chatter SLD for robot milling, it is essential to acquire the milling force coefficients for the ball-end milling cutter and the modal parameters of the robot system. By establishing a milling coefficient identification model based on the average milling force model, successful identification of the milling force coefficient for the ball-end milling cutter was achieved through a grooving experiment^28^.

**Figure 3:**
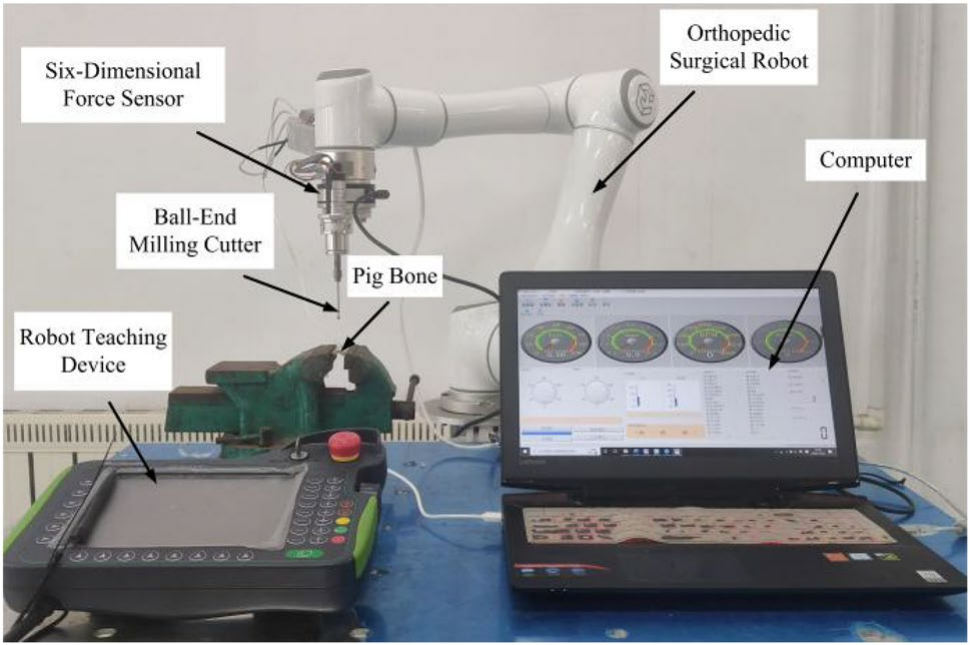
Robotbonemilling experimental setup.

The experimental procedure was conducted utilizing the experimental apparatus depicted in Fig. 3, comprising primarily an orthopedic surgical robot, a six-dimensional force sensor, a bone grinding drill, and a force sensor data acquisition system. The orthopedic surgical robot employed in the study is identified as the EC66 model, characterized by a maximum effective load capacity of  kg, a maximum end movement velocity of 2.8 m/s, a working radius spanning 914 mm, and a joint movement range spanning from − 360°to + 360°. The six-dimensional force sensor employed is designated as the KWR75B model, boasting a resolution capability of up to 0.03%FS. It is adept at capturing a maximum force magnitude of 200 N and a maximum torque of 8 Nm across the *x*, *y*, and *z* spatial axes. Throughout the experimental phase, the data sampling frequency of the six-dimensional force sensor was set to 1000 Hz, facilitating real-time collection of milling force data via the data acquisition system. For precise data acquisition, the six-dimensional force sensor was affixed to the tool mounting end of the robotic arm, with the bone grinding drill affixed to the sensor along the *Z*-axis of the robotic tool. To expedite the measurement of milling forces across the *x*, *y*, and z spatial axes, and to effectuate the completion of milling coefficient identification, the robotic arm executed a pig bone milling experiment in a vertical orientation, as delineated in Fig. 3.

The specific values obtained are presented in Table 1. During the operational phase of the orthopedic surgery robot, the tip of the ball-end milling cutter comes into direct contact with the bone. To obtain the milling modal parameters at the cutter's tip, a modal hammering experiment is conducted in this section.

**Table 1:** Milling force coefficients for ball end milling cutter.

*K*_*tc*_(N/mm^2^)	*K*_*rc*_ (N/mm^2^)	*K*_*ac*_ (N/mm^2^)
375.61	380.12	315.72

**Figure 4:**
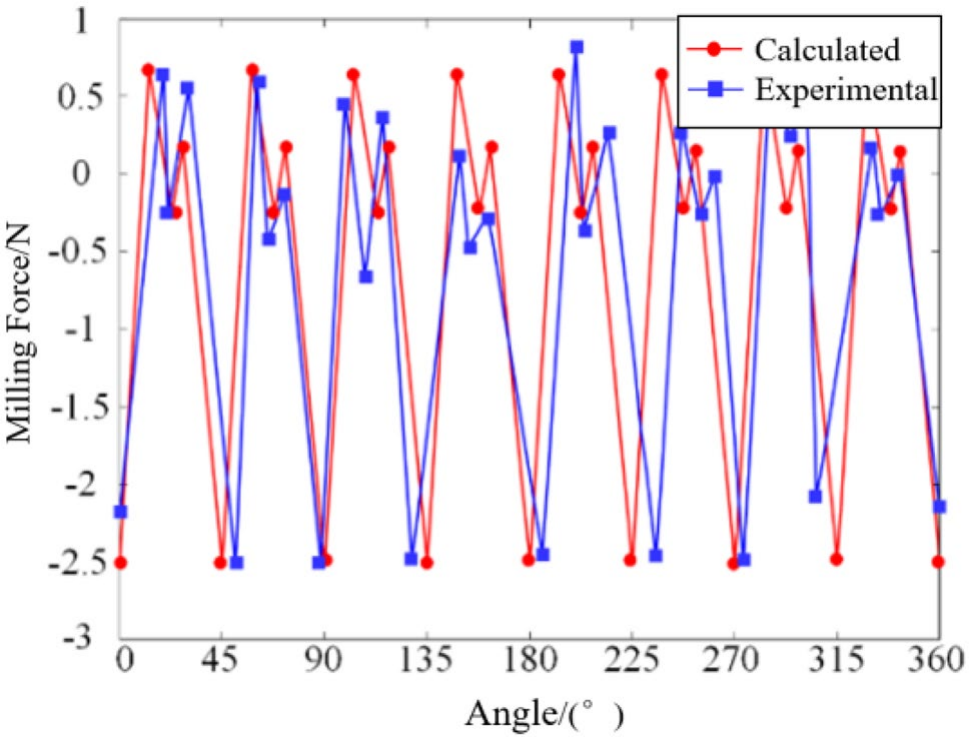
Comparison diagram of milling force in *X* direction.

**Figure 5:**
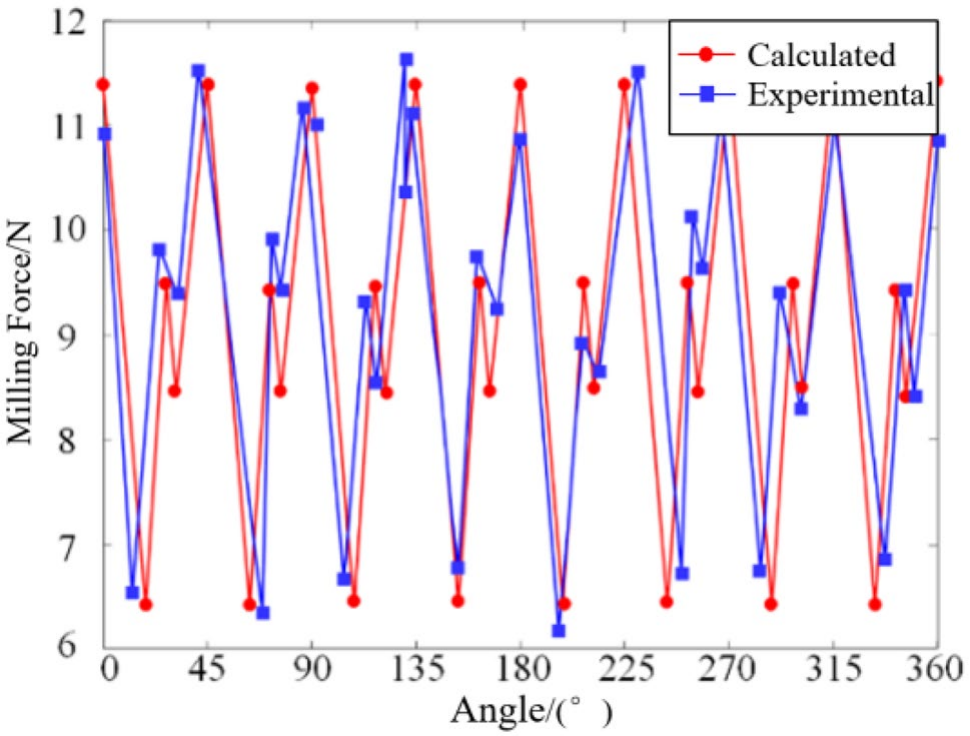
Comparison diagram of milling force in *Y* direction.

**Figure 6:**
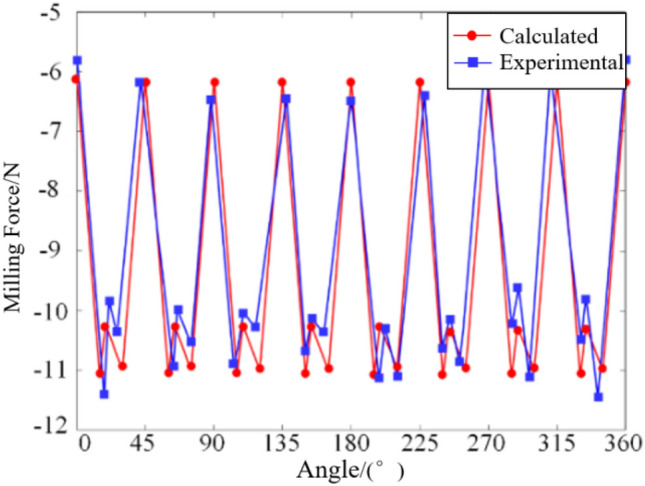
Comparison diagram of milling force in *Z* direction.

Through the milling force coefficient identification experiment, a comprehensive milling force model for the ball-end milling cutter was derived. To validate the accuracy of the established milling force model and the experimentally derived milling force coefficients, it is imperative to ascertain the trajectory of the milling force model and juxtapose it against the averaged results from five actual measured milling forces. In this regard, the selection of pertinent milling parameters common to orthopedic surgery was essential, encompassing spindle speed ($n{\kern 1pt} = 5000 r/\min$), milling depth (*a*_*p*_ = 0.5 mm), feed speed (*V*_*f*_ = 60 mm/min), and the corresponding milling coefficients in Table1. The graphical representations depicting the comparison of milling forces in each direction, as obtained from simulation and experimentation, are delineated in Figs. 4, 5, and 6.

The graphical representation elucidates a congruence between the calculated milling force curve and its experimental counterpart in terms of both magnitudes and evolving trends. Nonetheless, discernible deviations persist between the two curves, with maximum disparities observed in the milling forces along the *X*, *Y*, and *Z* directions amounting to 0.5 N, 0.8 N, and 0.6 N, respectively. These deviations stem primarily from data acquisition errors attributable to factors such as vibrational interferences during the milling operation and deviations in the tilt angle of the milling cutter falling short of or exceeding the predefined threshold. Despite these discrepancies, the average and peak values derived from both curves exhibit notable stability. Consequently, it can be deduced that the established milling force model, along with the associated milling force coefficients, remains reliable and precise within an acceptable margin of error.

### Modal experiment

The vibration system of the robot can be considered as a composition of multiple single-degree-of-freedom systems. The displacement frequency response function of the robot is defined as the ratio of the amplitude of the steady-state displacement response to the amplitude of the simple harmonic excitation, expressed as follows.

12 $$H\left( \omega \right) = \frac{X}{F} = \frac{1}{{ - \omega^{2} m + i\omega c + k}}$$

where $F$ is the amplitude of the simple harmonic excitation,$X$ is the amplitude of the steady-state displacement response,$\omega$ is the frequency, *m*, *c*, and *k* represent the modal mass, modal damping, and modal stiffness, respectively.

By introducing the undamped natural frequency $\omega_{n} = \sqrt {k/m}$, damping ratio $\xi = c/2\sqrt {mk}$, and frequency ratio $\lambda = \omega /\omega_{n}$, the displacement frequency response function can be expressed as follows:

13 $$H\left( \omega \right) = \frac{1}{{k\left( {1 - \lambda^{2} + 2i\xi \lambda } \right)}}$$

**Figure 7:**
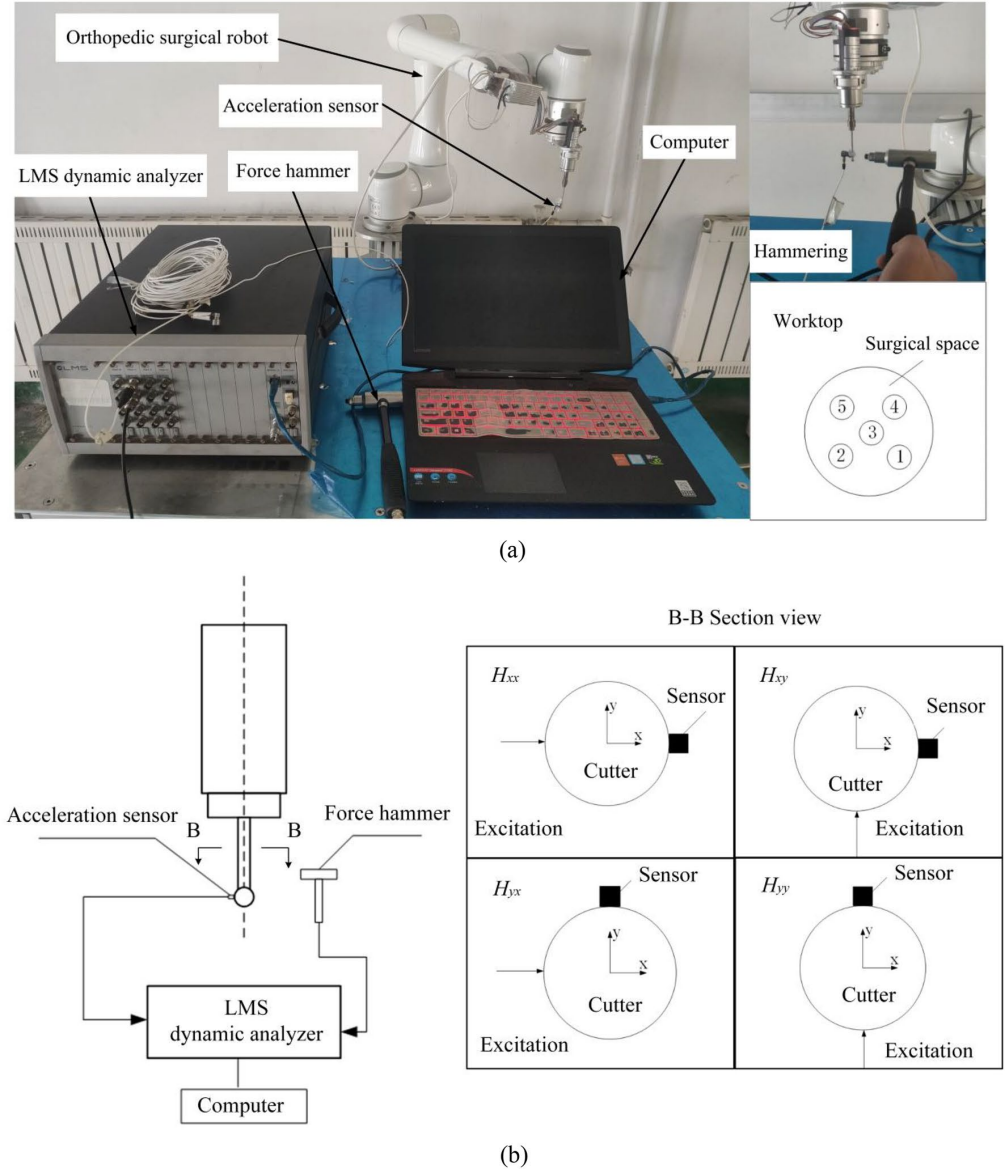
Modal experiment procedures. Where (a) Force hammer experiment setup and (b) Force hammer experiment with different directions.

The extraction of natural frequency $\omega_{n}$, modal stiffness *k*, and damping ratio *ξ* for the frequency response function at the tip of the cutter requires the implementation of modal experiments, as evident from the aforementioned equations. The modal experiment procedures are elaborated in Fig. 7.

The modal hammering experiment is conducted on the EC66 orthopedic robot, characterized by a load capacity of 6 kg and a repeat positioning accuracy of ± 0.2 mm, with the milling head installed at the robot's end (Fig. 7a). Given that fixing the acceleration sensor onto the milling cutter may influence the intrinsic frequency of the cutter tip due to the coupling between the mass and stiffness of the sensor and the milling cutter, measures are taken to mitigate this effect. Specifically, a lightweight and low-stiffness sensor, the 353B15 acceleration sensor, is selected and positioned as close as possible to the tool tip. The sensor features a sensitivity of 10.44mv/m/s^2^, a resolution of 0.005 grms, a range of 500 gpk, and weighs 2 g. Additionally, the suspended length of the milling cutter is set to 40 mm. To induce impact on the cutter, a PCB impact hammer is utilized, boasting a measurement range of ± 444 N pk and a force sensor sensitivity of 11.2 mV/N. The excitation signal is recorded and processed using the LMS dynamic analyzer, with resulting vibration signals analyzed through the modal analysis module of the LMS Test Lab analysis software. This analysis provides time domain signals, frequency domain signals, and coherence functions to ascertain the modal parameters of the robot.

Orthopedic robots frequently engage in surgeries necessitating proximity to the surgical target, maintaining a fixed orientation while adjusting their position in tandem with the surgical target's movements throughout the procedure. Given the variability in the robot's position, its modal parameters undergo corresponding changes, underscoring the importance of analyzing the robot's stability across various surgical positions. In this study, we focus on the workspace of the robot during vertebral plate cutting surgery, wherein five distinct positions within the surgical workspace are delineated, as illustrated in Table 2. This analysis aims to elucidate the impact of modal parameter variations on the stability of robot milling operations.

**Table 2:** Modal experimental joint angle under fixed attitude(unit:°).

Positions	*θ*_1_	*θ*_2_	*θ*_3_	*θ*_4_	*θ*_5_	*θ*_6_
1	4.88	− 90.46	100.51	258.22	90.45	141.44
2	80.19	− 75.73	96.51	248.86	88.38	218.40
3	36.91	− 84.20	100.39	251.16	91.03	169.77
4	11.81	− 72.57	77.67	263.17	88.67	150.59
5	60.38	− 72.65	79.45	262.91	89.03	197.86

In the experimental configuration (Fig. 7b), the force hammer is utilized to individually excite the cutter along the *x*-direction and *y*-direction, with multiple excitations performed in each direction. The hammering process is meticulously assessed by scrutinizing the excitation signals and responses. If necessary, re-hammering is conducted to ensure a minimum of five valid excitations in each direction, and the average values are subsequently employed.

The collected data undergoes truncation, whereby the first 40 data points of the excitation response are retained. Subsequently, the time domain data is subjected to Fast Fourier Transform (FFT) to effectuate the transformation into the frequency domain, thereby generating frequency domain plots and coherence function curves at each position. Based on the outcomes derived from these analyses, the modal frequencies of the robot are initially discerned.

In the process of modal experiment, there are more factors interfering with the experiment, in order to ensure the accuracy of the experimental results, the coherence function is also needed to be used as an evaluation index to assess the good or bad test results, and its expression is:

14 $$C_{xy} (k) = \frac{{\left| {S_{xy} (k)} \right|^{2} }}{{S_{xx} (k)S_{yy} (k)}}$$

where $S_{xx} {(}k{)}$ is the self-power of the excitation signal and the corresponding signal;$S_{yy} {(}k{)}$ is the mutual power of the excitation signal and the corresponding signal;$S_{xy} {(}k{)}$ is the spectral density function of the excitation signal and the corresponding signal.

The coherence function characterizes the relationship between input and output, meaning that how much of the input energy causes the corresponding output, and the closer the value of the coherence function is to 1, the higher the degree of coherence and the closer it is to the desired expectation. In general, a value of the coherence function greater than 0.8 indicates good coherence, and if its value is lower than 0.8, the data here is not available.

### Results of modal parameter identification for robot milling system

**Figure 8:**
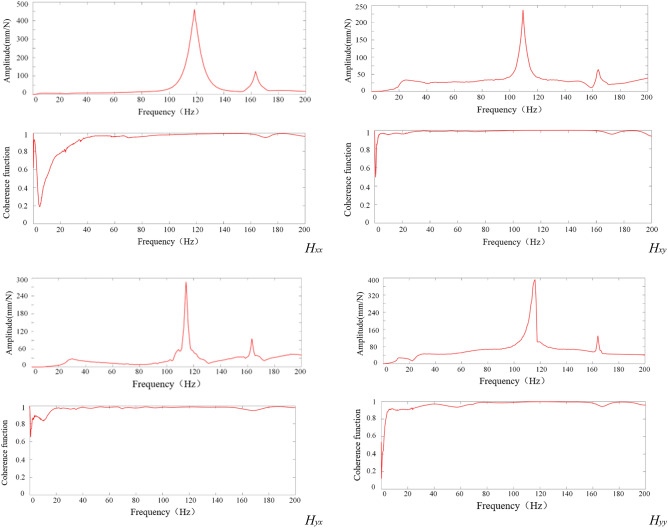
Frequency domain diagrams and coherence function curve for *H*_*xx*_, *H*_*xy*_, *H*_*yx*_ and *H*_*yy*_*.*

As an illustration, the hammering experiment was conducted at position 3 to obtain the time-domain diagram. The time-domain diagram was then transformed into a frequency-domain diagram using FFT processing and analysis. Based on the converted results, the frequency domain diagrams and coherence function curve at position 3 were obtained, as depicted in Fig. 8.

The half-power bandwidth method is a method commonly used to calculate the damping ratio of a structure, especially in modal analysis. The method is based on the frequency response curve of the structure and estimates the damping ratio by measuring the half-power point (i.e., the point of 0.707 times the amplitude with respect to the peak value) near the natural frequency of a particular mode. The peak frequency of one of the modes of the structure is found by modal analysis or spectral analysis, i.e., the natural frequency $\omega_{n}$ is measured. Find points on either side of the peak frequency that are 0.707 times the amplitude relative to the peak value *H*_*m*_. These two points correspond to the half-power bandwidth near the natural frequency. The half-power bandwidth $\Delta \omega$ is calculated by measuring the frequency difference between the half-power points. The damping ratio $\xi$ can be calculated by dividing the half-power bandwidth by twice the natural frequency.

**Figure 9:**
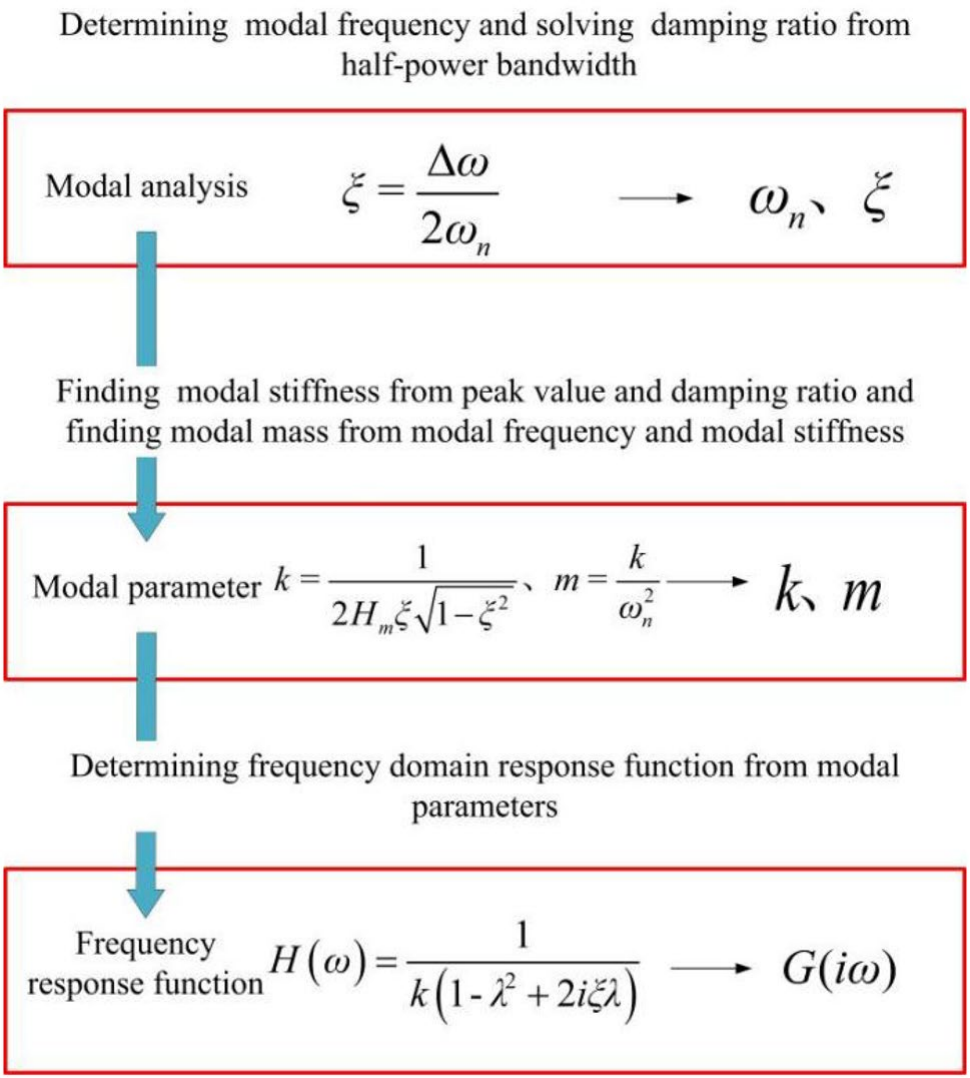
Flow chart of modal parameter identification.

Following the flow chart in Fig. 9 for modal parameter identification, the modal parameters of the orthopedic surgery robot during the milling process at position 3 were determined. Similarly, the same process was applied to the other positions, resulting in the acquisition of modal parameters for the robot at different milling positions. The corresponding outcomes are presented in Table 3. Notably, Table 3 reveals that the dynamic characteristics at the tip of the ball end milling cutter vary depending on the robot's position during operation. Consequently, the stability of the robot dynamically changes throughout the milling process. Thus, the selection of an appropriate working position can enhance the milling performance of the robots in practical applications.

**Table 3:** Modal parameters of robots at different positions.

Directions	Positions	Modal frequency (Hz)	Damping ratio (%)	Modal stiffness (× 10^4^ N/m)
*xx*	1	116.71	2.647	3.24
	2	114.37	3.218	4.62
	3	118.42	2.196	5.05
	4	120.64	3.623	3.14
	5	115.25	2.334	3.59
*xy*	1	110.21	2.232	3.12
	2	116.73	2.215	5.62
	3	116.23	2.363	4.12
	4	121.46	2.825	3.58
	5	117.53	2.132	3.56
*yx*	1	114.32	2.323	3.24
	2	115.48	3.235	5.12
	3	117.25	2.456	4.35
	4	121.35	3.359	3.56
	5	117.76	2.183	3.34
*yy*	1	113.49	2.157	3.12
	2	117.48	2.957	5.22
	3	115.23	2.612	3.96
	4	122.34	3.294	4.36
	5	118.76	1.983	3.55

**Figure 10:**
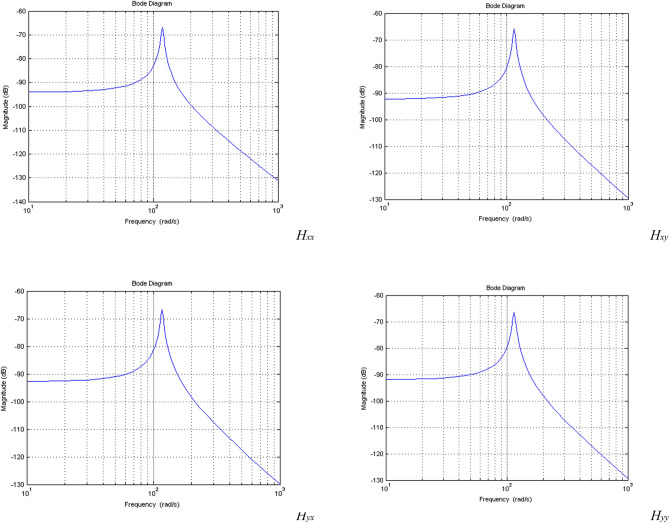
Frequency domain bode diagrams for *H*_*xx*_, *H*_*xy*_, *H*_*yx*_ and *H*_*yy*_*.*

Modal parameters delineated for each specified direction, as outlined in Table 3, have been computed utilizing Eq. (13). This computation facilitated the generation of Bode plots for the respective frequency domain response functions, with outcomes presented in Fig. 10. Upon conducting a comparative analysis, it is observable that the frequency domain response functions associated with the modal parameters for each direction, as depicted in Fig. 8, exhibit fundamental concordance with the experimentally measured frequency domain response functions illustrated in Fig. 10. Such concordance substantiates the efficacy and applicability of the modal parameter estimation methodology introduced in this paper.

## Experimental validation of chatter stability lope diagram

**Figure 11:**
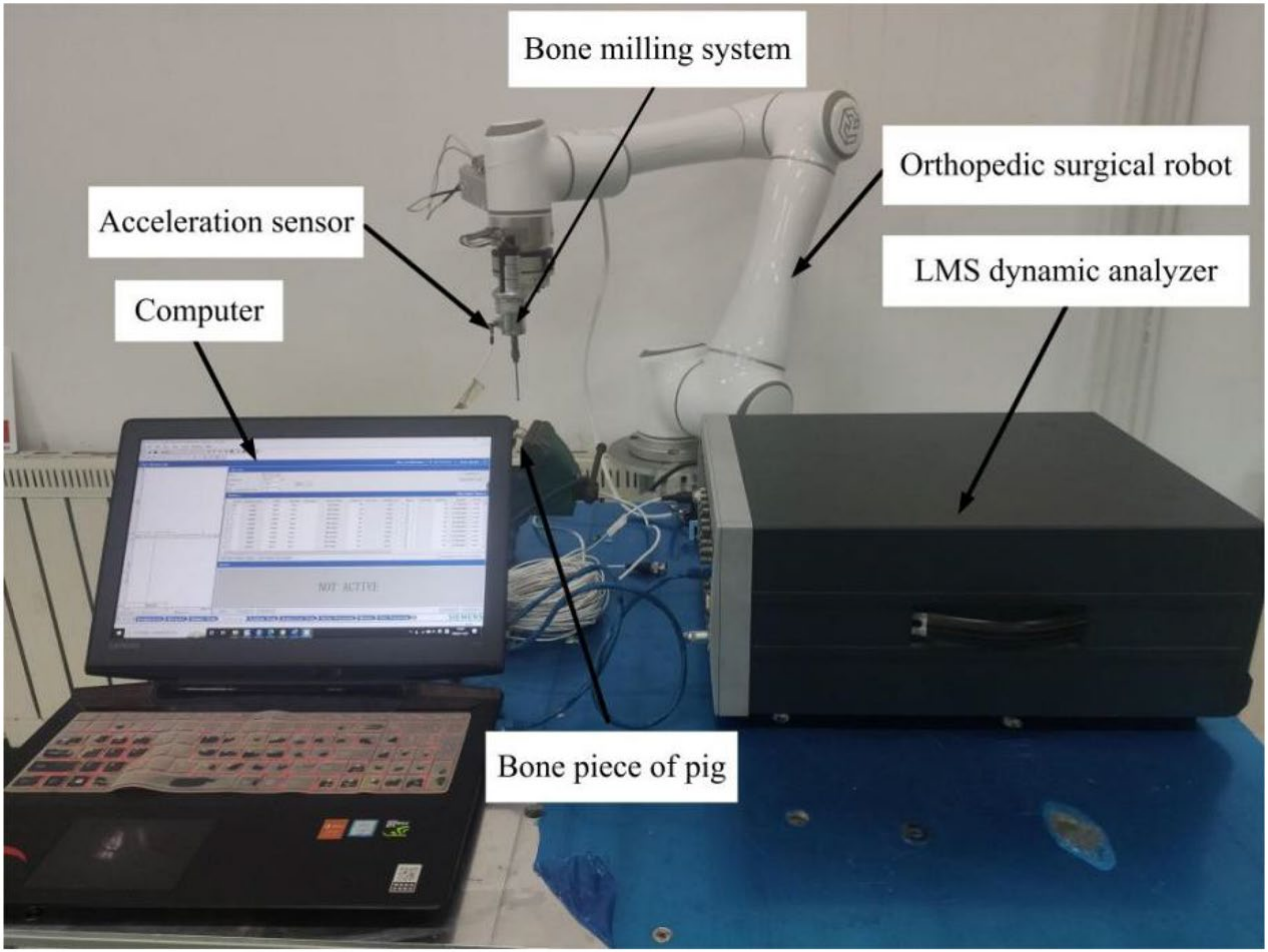
Experimental setup for milling chatter stability of orthopedic surgery robot.

To validate the accuracy of the chatter SLD, an experimental verification was conducted. The experimental setup for the robot chatter stability experiment included an orthopedic surgery robot, a six-dimensional force sensor, a bone milling system, an acceleration sensor, and an LMS dynamic analyzer (Fig. 11). The acceleration sensor was mounted on the milling cutter base shell, and the robot demonstrator was used to program and plan the milling path. The milling conditions, such as spindle speed, feed rate, milling depth, and robot position, were continuously adjusted while monitoring milling force changes in real-time using the six-dimensional force sensor. Simultaneously, acceleration sensors collected vibration signals during the milling process, which were processed and stored in a computer. Chatter conditions during milling were determined by analyzing these signals. The medical ball-end milling head is installed on the robot end. A eight-tooth carbide milling cutter with a diameter of 4 mm is fixed in the cutter holder. Pig leg bone was used as the material for the experiment.

Prior to the experiment, the orthopedic surgery robot was positioned to correspond to working position. To minimize deviations in the robot's movement relative to working position, a radial milling depth of 5 mm was set, and the axial milling position angle in the experiment was aligned with that used in stability calculations. The milling feed rate was directed along the positive *Y*-axis, and a smooth milling method was employed. The acceleration sensor, placed near the spindle, monitored milling vibration changes by collecting acceleration signals during the process, enabling the identification of chatter conditions. The milling parameters used during the experiment are listed in Table 4.

**Table 4:** Milling parameters.

Serial number	Rotation speed(r/min)	Axial milling depth (mm)	Radial milling depth (mm)
1	4000	0.2	5
2	4000	0.4	5
3	5000	0.2	5
4	5000	0.4	5
5	6000	0.2	5
6	6000	0.4	5
7	7000	0.2	5
8	7000	0.4	5

**Figure 12:**
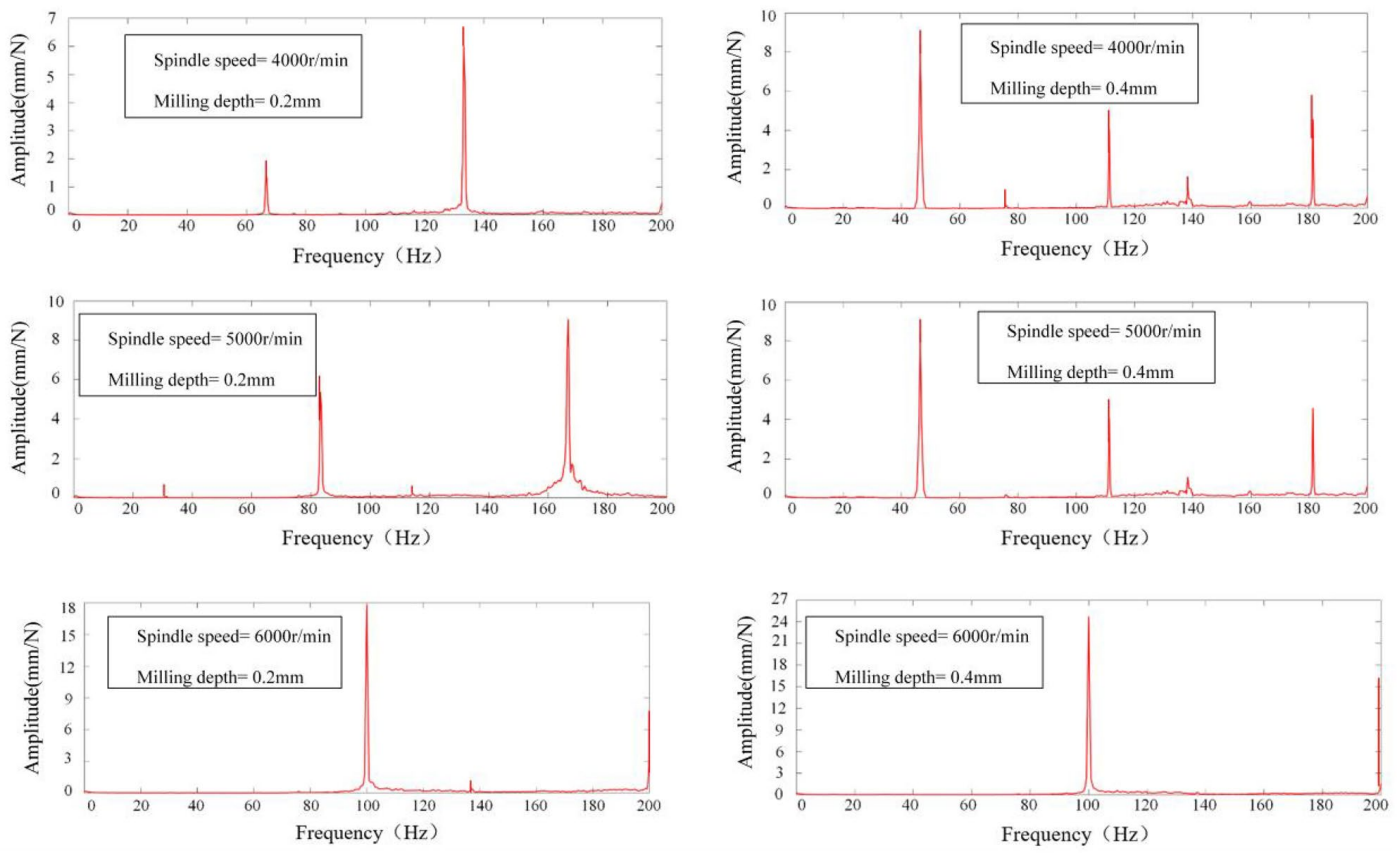
Spectrum diagram under different milling parameters at Position 1.

**Figure 13:**
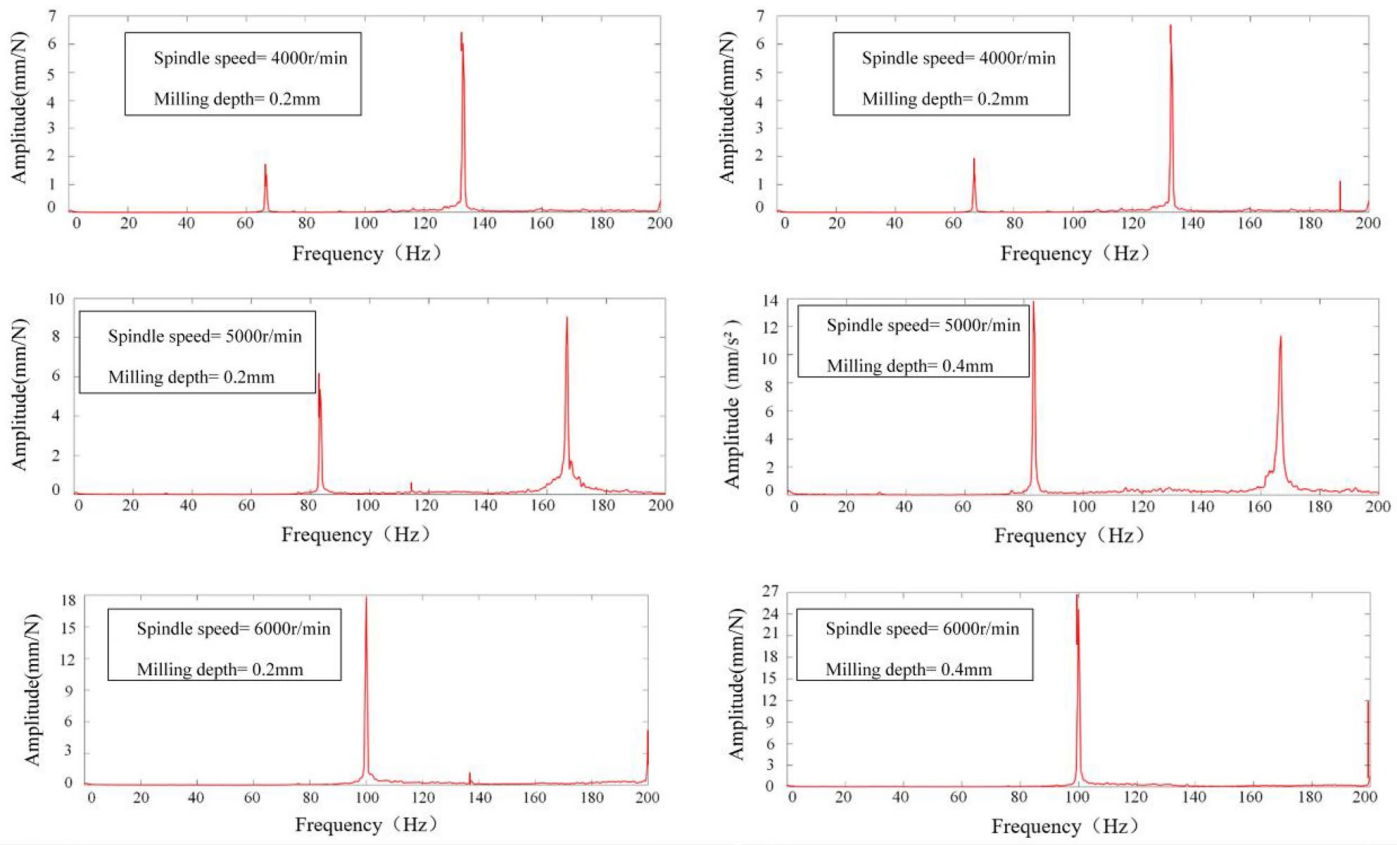
Spectrum diagram under different milling parameters at Position 2.

**Figure 14:**
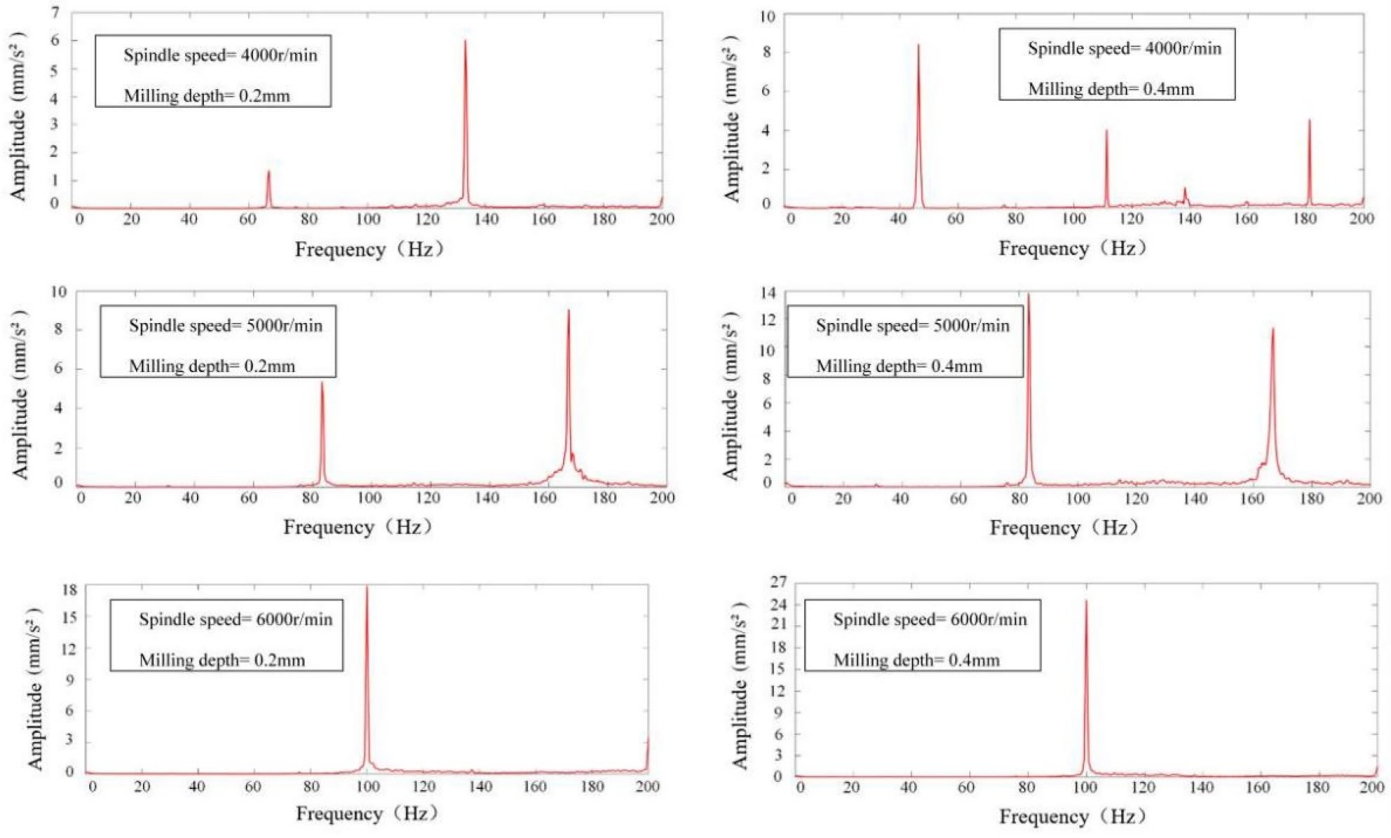
Spectrum diagram under different milling parameters at Position 3.

**Figure 15:**
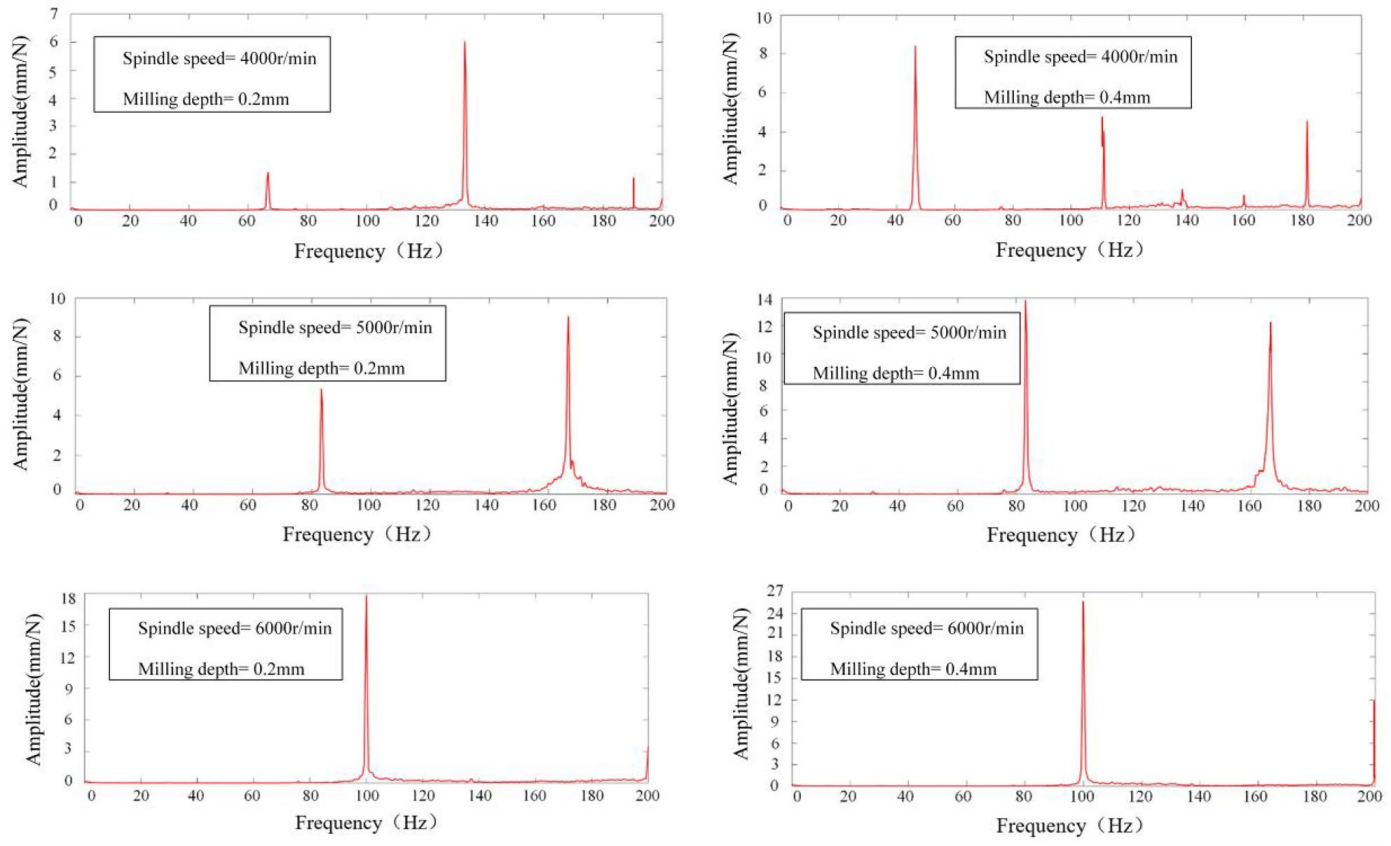
Spectrum diagram under different milling parameters at Position 4.

**Figure 16:**
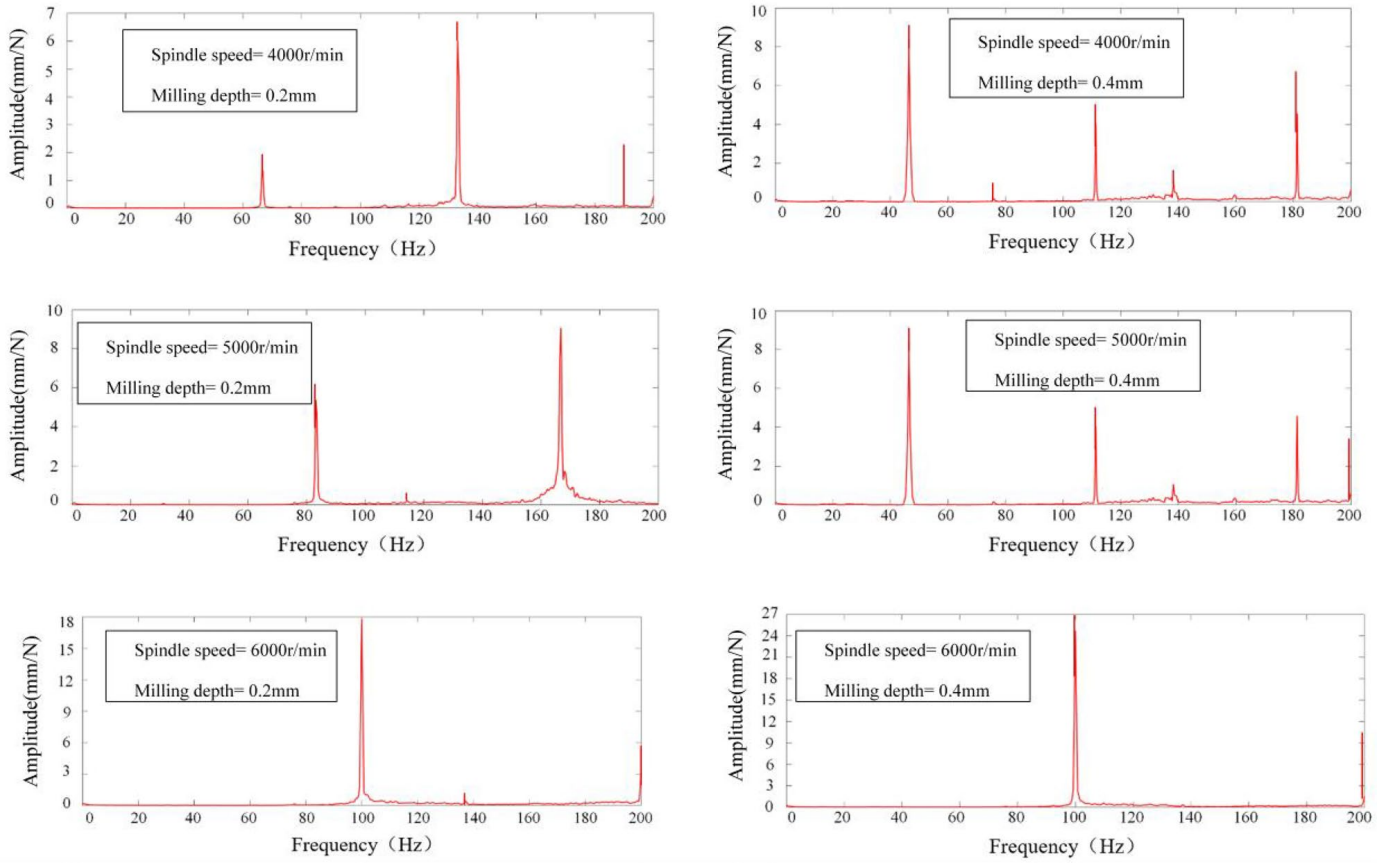
Spectrum diagram under different milling parameters at Position 5.

The vibration signals acquired were subjected to a Fast Fourier Transform (FFT) analysis within Matlab to elucidate the frequency domain characteristics of the milling process. This spectral analysis facilitated the discernment of chatter phenomena during the operation. The determination of chatter presence was based on the location of the peak within the signal's power spectrum's horizontal axis. Specifically, for spindle speeds of 4000, 5000, and 6000 revolutions per minute (rpm), the spindle rotation frequencies (*SF*) at positions 1–5 were delineated in Table 5. Fourier transformation of the acquired signals from positions 1 to 5 yielded spectrograms, depicted in Figs. 12, 13, 14, 15 and 16, which were instrumental in identifying instances of chatter. A peak frequency in the signal spectrum aligning closely with an integer multiple of the spindle frequency indicated the absence of chatter. In contrast, a deviation from this pattern was indicative of the occurrence of chatter within the system.

**Table 5:** Spindle rotation frequency at positions 1 to 5.

Positions	Rotation speed, *n*(r/min)	Spindle rotation frequency, *SF*(Hz)
1–5	4000	66.79
	5000	83.21
	6000	100

**Figure 17:**
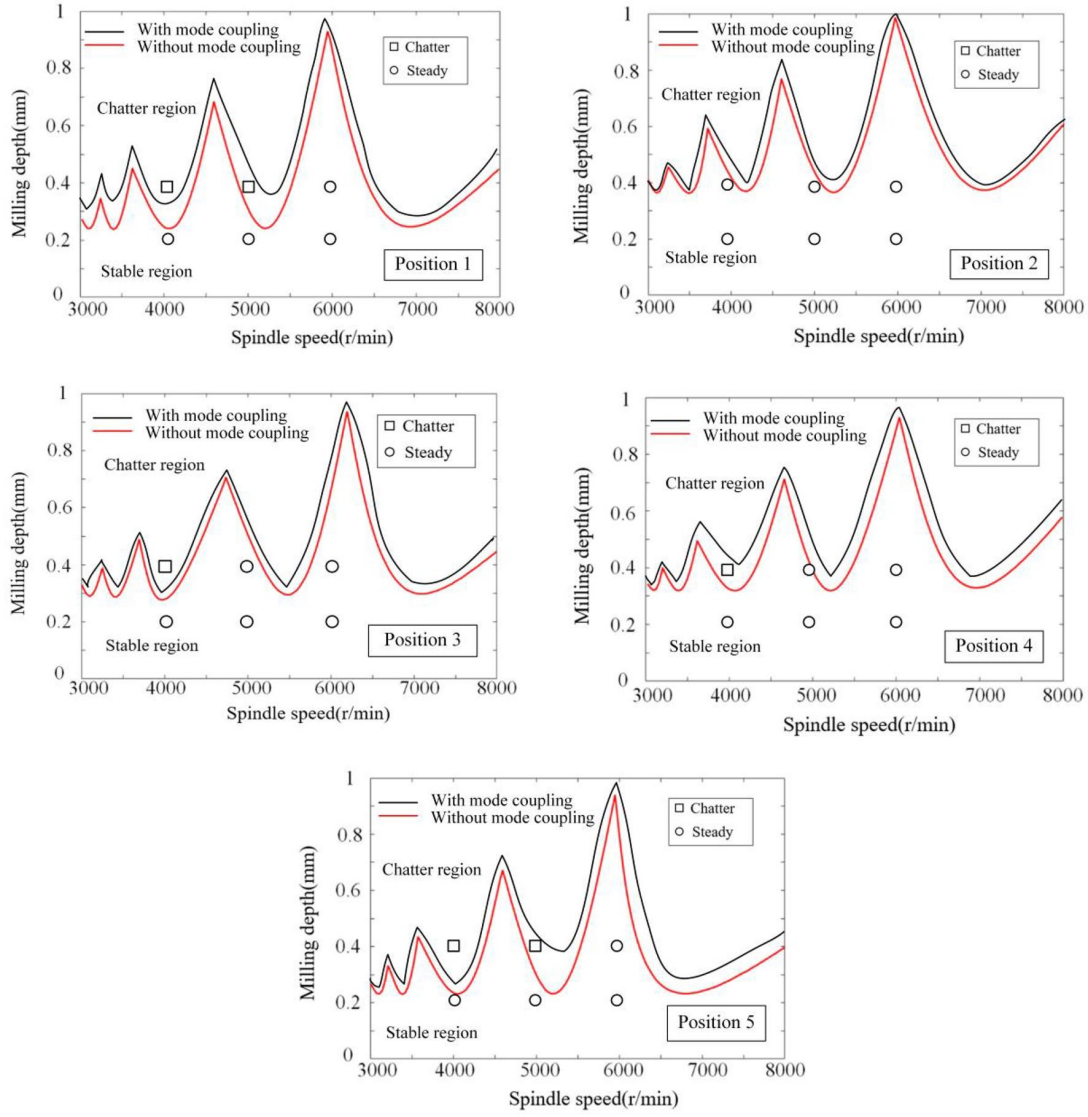
SLD curves at position1–5 of different parameters.

Owing to the inherently low structural rigidity characteristic of serial robots, the stability of the robot milling system is influenced by both regenerative chatter and mode coupling chatter. As illustrated in Fig. 17, the SLD curves demarcate the parameter space for milling into a chatter zone (located above the curve) and a stable zone (situated below the curve). The black curve represents the SLD curve derived from an analysis that incorporates both the mode coupling and regenerative effects, whereas the red curve corresponds to the SLD curve generated with exclusive consideration of the regenerative effect. It is manifest that the inclusion of the mode coupling effect in the analysis significantly expands the stability region within the milling process context.

Milling experiments were conducted in accordance with the parameters delineated in Table 4, correlating to the marked points at positions 1–5 under the designated milling conditions as depicted in Fig. 17. Based upon the SLD curves prognosticated in Fig. 17, it is discernible that the conditions specified—namely, Spindle Speed at 4000 rpm and Milling Depth at 0.4 mm for Positions 1 and 5, as well as Spindle Speed at 5000 rpm and Milling Depth at 0.4 mm at Positions 1 and 5, in addition to Spindle Speed at 4000 rpm and Milling Depth at 0.4 mm at Positions 3 and 4—predominantly fall within the chatter domain. Conversely, only the conditions of Spindle Speed at 5000 rpm and Milling Depth at 0.4 mm at Positions 1 and 5, alongside Spindle Speed at 4000 rpm and Milling Depth at 0.4 mm at position 4, are ascertained to be within the stable domain, this determination taking into account both the mode coupling effect and the regenerative effect. When specifically accounting for the mode coupling effect, all other marked points are deemed to reside within the stable domain. The spectrograms of signals from positions 1 to 5, as exhibited in Figs. 12, 13, 14, 15 and 16, along with the SLD curves for positions 1 to 5 under various parameters showcased in Fig. 17, exhibit consistent correlations between stable and chatter states. This consistency underscores the accuracy of the SLD analysis methodology employed in this study, affirming the utility of the derived chatter SLD curves for conducting milling chatter stability analysis.

## Influence of modal parameters on milling chatter stability

This section aims to investigate the influence of various modal parameters on chatter stability using the control variable method. Each modal parameter's influence is examined by varying the natural frequency, damping ratio, and modal stiffness while keeping the other parameters constant.

### Influence of natural frequency on chatter stability

During the modal experiments, the natural frequency of the milling system ranged from 114 to 121 Hz in the *x*-direction and from 113 to 123 Hz in the *y*-direction for the robot at its five positions. The differences between the maximum and minimum natural frequency values in both directions were 6.14% and 8.85%, respectively.

**Figure 18:**
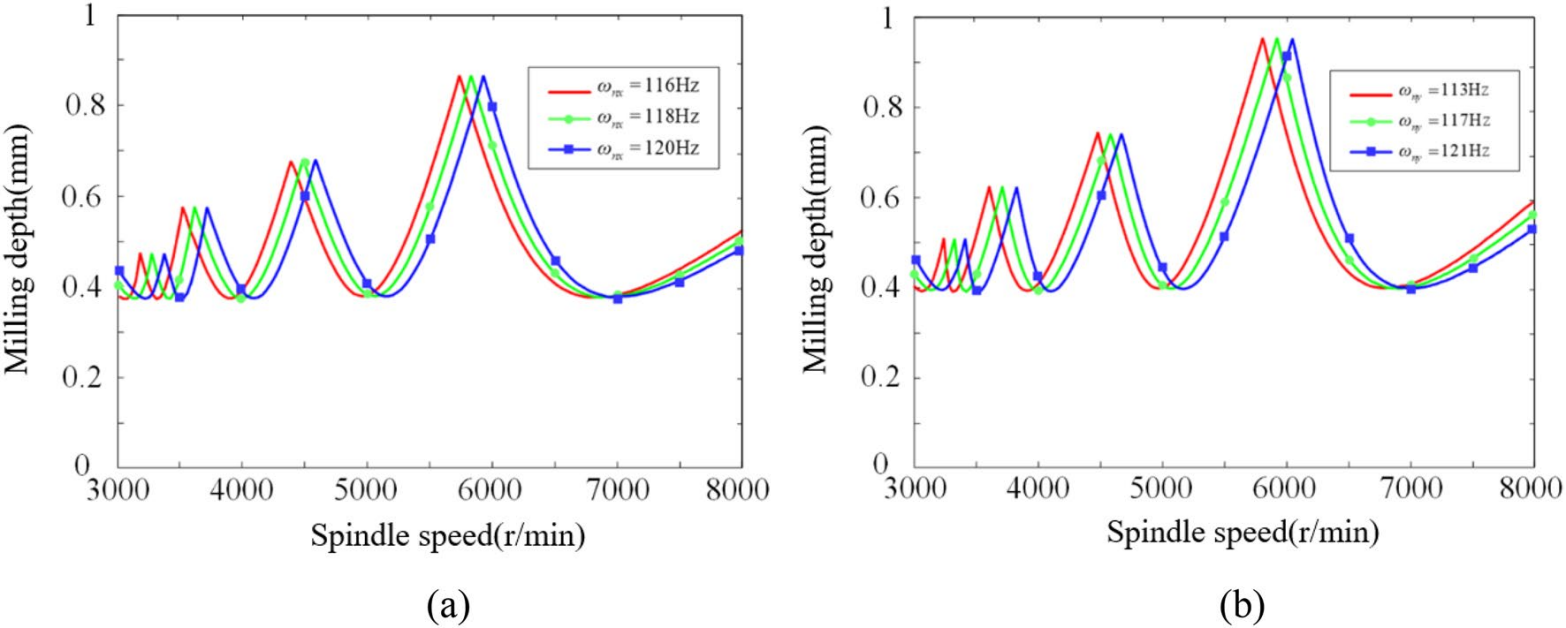
Influence of *x*-direction and *y*-direction natural frequency on chatter stability. Where (a) x-direction and (b) y-direction.

To investigate the influence of natural frequency on chatter stability in the *x*-direction, while keeping the damping ratio and modal stiffness constant, the natural frequencies in the *x*-direction were varied (116 Hz, 118 Hz, and 120 Hz) while maintaining a natural frequency of 113 Hz in the *y*-direction. The resulting chatter SLD is shown in Fig. 18a. To investigate the influence of natural frequency on chatter stability in the *y*-direction, while keeping the damping ratio and modal stiffness constant, the natural frequency in the *x*-direction was set to 116 Hz, while the natural frequencies in the *y*-direction were varied (113 Hz, 117 Hz, and 121 Hz). The corresponding chatter SLD is depicted in Fig. 18b.

From Fig. 18, it can be observed that changing the natural frequencies of the milling system does not alter the shape of the chatter SLD, and the peak and valley values remain consistent. Increasing the natural frequency of the milling system results in an overall rightward shift of the chatter SLD. However, while this shift changes the position of the diagram, it does not affect the size of the chatter stability region. In other words, increasing the natural frequency does not enhance the system's stability. When chatter occurs during the milling process, adjusting the system's natural frequency to position it within the chatter stability region may help improve stability.

### Influence of damping ratio on chatter stability

During the modal experiments, the damping ratio of the milling system varied from 2.1 to 3.7% in the *x*-direction and from 1.9 to 3.3% in the *y*-direction across the five positions. The differences between the maximum and minimum damping ratio values in both directions were 1.6% and 1.4%, respectively. To investigate the influence of the damping ratio on chatter stability at different damping ratios, the damping ratios in the *x*-direction and *y*-direction were varied within their respective ranges.

**Figure 19:**
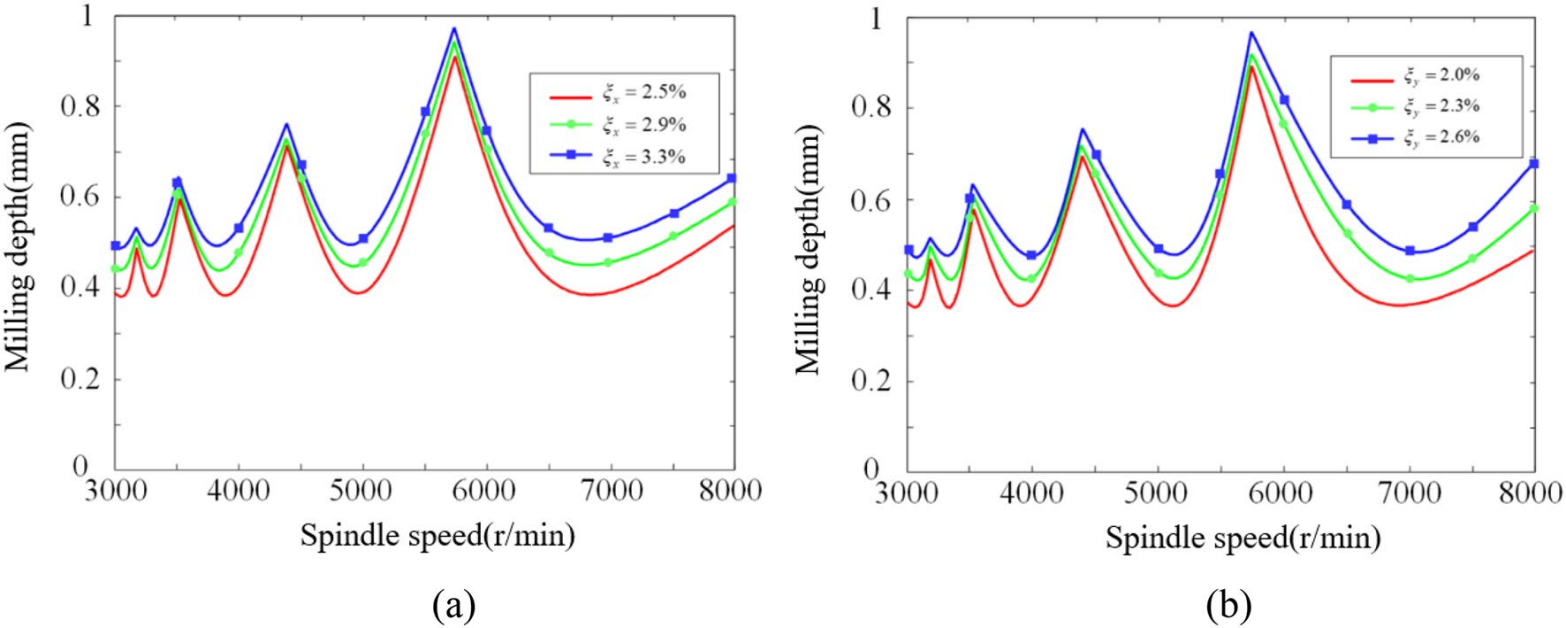
Influence of damping ratio in *x*-direction and *y*-direction on chatter stability. Where (a) x-direction and (b) y-direction.

In the study of the influence of damping ratio on chatter stability in the *x*-direction, while keeping the natural frequency and modal stiffness constant, the damping ratios in the *x*-direction were set to 2.5%, 2.9%, and 3.3%, while maintaining a damping ratio of 2.0% in the *y*-direction. The resulting chatter SLD is shown in Fig. 19a. In the study of the influence of damping ratio on chatter stability in the *y*-direction, while keeping the natural frequency and modal stiffness constant, the damping ratios in the *y*-direction were set to 2.0%, 2.3%, and 2.6%, while maintaining a damping ratio of 2.5% in the *x*-direction. The corresponding chatter SLD is depicted in Fig. 19b.

From Fig. 19, it can be observed that increasing the damping ratios in both the *x*-direction and *y*-direction has a similar influence on the robot's stability. As the damping ratios increase, the curves of the chatter SLD shift upward, resulting in an expanded milling stability region. A higher damping ratio allows the robot milling system to absorb chatter energy more effectively, enhancing stability. Although both the peaks and valleys of the chatter SLD shift upward with increasing damping ratios, the displacement of the valleys is larger than that of the peaks. To achieve higher stability during the milling process, selecting a higher damping ratio is recommended. Additionally, if the milling system has a high damping ratio, the milling depth can be increased appropriately to improve efficiency by increasing the rate of bone removal.

### Influence of modal stiffness on chatter stability

During the modal experiments, the modal stiffness of the milling system varied from 3.1 × 104 to 5.1 × 104 N/m in the *x*-direction and from 3.2 × 104 to 5.3 × 104 N/m in the *y*-direction across the five positions. The differences between the maximum and minimum modal stiffness values in both directions were 2 × 104 N/m and 2.1 × 104 N/m, respectively. To investigate the influence of modal stiffness on chatter stability under different modal stiffness values, the modal stiffness values were varied within the corresponding ranges.

**Figure 20:**
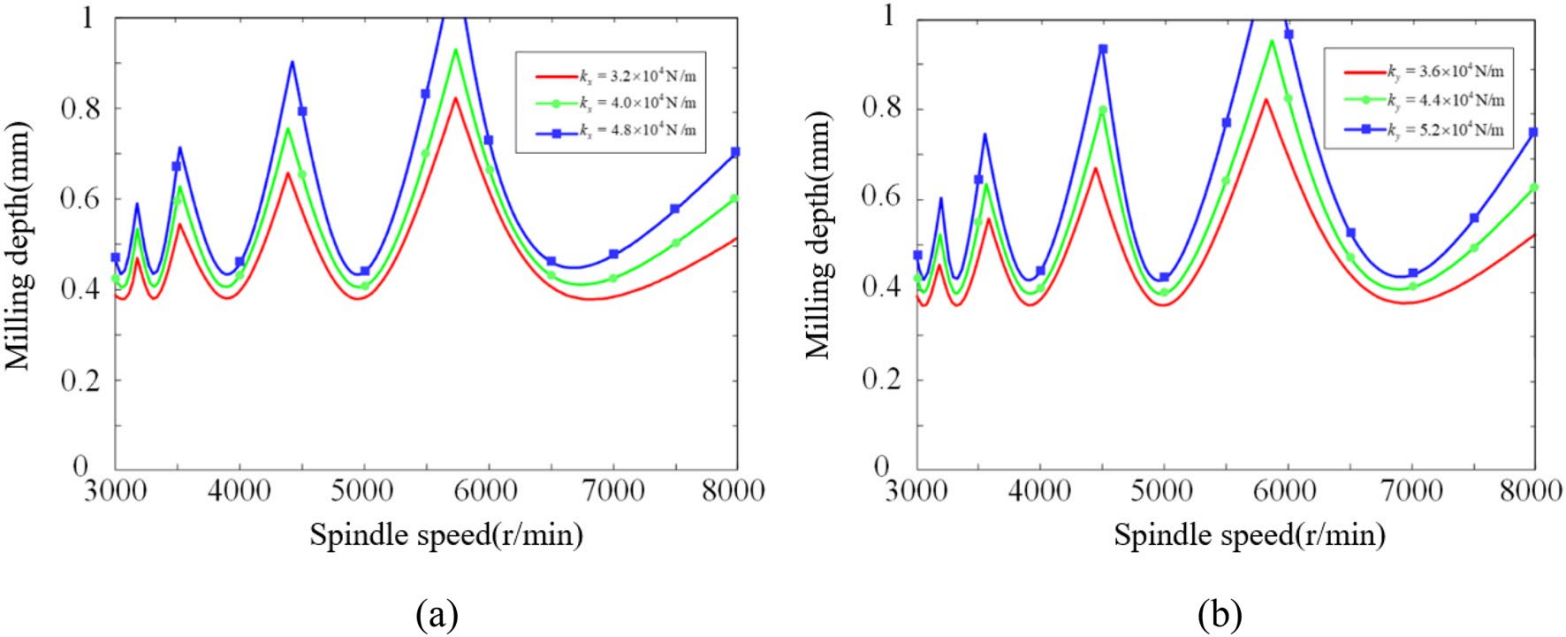
Influence of *x*-direction and *y*-direction modal stiffness on chatter stability. Where (a) x-direction and (b) y-direction.

In investigating the influence of modal stiffness on chatter stability in the *x*-direction, while keeping the natural frequency and damping ratio constant, the modal stiffness values in the *x*-direction were set to 3.2 × 104 N/m, 4.0 × 104 N/m, and 4.8 × 104 N/m, while maintaining a modal stiffness of 3.6 × 104 N/m in the *y*-direction. The resulting chatter SLDs are shown in Fig. 20a. In investigating the influence of modal stiffness on chatter stability in the *y*-direction, while keeping the natural frequency and damping ratio constant, the modal stiffness values in the *y*-direction were set to 3.6 × 104 N/m, 4.4 × 104 N/m, and 5.2 × 104 N/m, while maintaining a modal stiffness of 3.6 × 104 N/m in the x-direction. The corresponding chatter SLDs are depicted in Fig. 20b.

From Fig. 20, it can be observed that increasing the modal stiffness in both the *x*-direction and *y*-direction has a similar influence on the robot's stability. As the modal stiffness increases, the overall curve of the chatter SLD shifts upward, resulting in an expanded milling stability region. During the upward shift of the chatter stability curve, both the peaks and valleys increase proportionally, maintaining a constant peak-to-valley ratio. Since the natural frequency of the robot milling system remains constant, the peaks and valleys of the chatter stability curve do not change in the horizontal position. Increasing the modal stiffness of the robot milling system can provide a larger stable region during the milling process. Therefore, selecting a region with higher modal stiffness, while keeping other parameters constant, can improve milling stability.

## Conclusion

This research investigates the impact of position, natural frequency, damping ratio, and modal stiffness on the milling chatter stability of an orthopedic surgery robot, accounting for both mode coupling and regenerative effects. The findings reveal that the chatter stability domain model formulated within this study is capable of predicting the stability of the robot milling system across varying milling parameters. The key contributions and conclusions of this research are articulated as follows:

1. Leveraging the modal coupled effect and the regenerative effect, this study developed a dynamic milling force model and a chatter stability domain model for the robotic milling process, employing the zero-order frequency domain method for its foundation.
2. A novel method for constructing chatter SLDs was introduced, offering a robust tool for assessing the stability of robotic operations during milling tasks.
3. Through the identification of experimental modal parameters at various positions, precise data was acquired for the generation of SLDs, enhancing the accuracy of stability predictions.
4. The reliability and effectiveness of the proposed chatter SLD analysis technique were corroborated through experimental validation, laying a solid groundwork for subsequent investigations into chatter stability across diverse modal parameters.
5. An analysis of the effects of different modal parameters on the stability of robot milling chatter was conducted, providing a theoretical framework for the selection of cutting parameters and the development of control strategies tailored to orthopedic surgery robots.

## Data Availability

All relevant data are within the paper.
